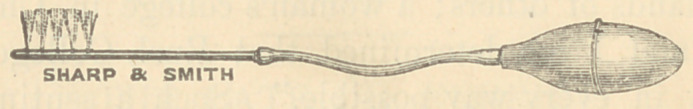# A Very Useful Nasal Irrigator

**Published:** 1877-05

**Authors:** Norman Bridge

**Affiliations:** Chicago


### A VERY USEFUL NASAL IRRIGATOR.

EDITOR JOURNAL AND EXAMINER:

Experience seems to have demonstrated that the nasal douche—while with proper care very useful in its way—in the hands of most patients and many practitioners, is a dangerous instrument, on account of the tendency of its use to cause congestion and inflammation of the middle ear. It is desirable to have some means of thoroughly irrigating the nasal passages in cases of catarrh and ozena, without the danger referred to. Different forms of syringe have been used with more or less success.

The curved syringe for injecting fluid forward from the posterior nares, is in some hands very successful; but fluid thrown back through the anterior nares sometimes strikes against the mouths of the eustachian tubes, and proves as mischievous as when coming through the douche under considerable pressure.

The little instrument figured below—and which has been made for me by Messrs. Sharp & Smith—has been contrived for the purpose of a nasal irrigator, and is believed to be free from the slightest danger of injuring the ear, while it has the advantage of being much easier of use than any syringe, and much more effectual. It consists of nothing but a rubber bulb, a short rubber tube and a metallic tube perforated on one side near its extremity, as shown in the cut.

The mode of using it is easily understood from its construction. The metallic tube is passed two inches or more into the nasal fossa, exactly as in passing a eustachian catheter, the perforations directed upward, when the bulb, previously filled with the irrigating fluid is forcibly compressed, and the fluid is projected against the roof of the fossa, exactly where irrigation is needed. The manipulation is repeated until a sufficient effect is accomplished. Patients are easily taught to use it. The metallic tube may be attached to an ordinary syringe bulb and a continuous stream of water ensured.

Of course there is no new principle employed in the construction of the instrument, and it may have been made and used in this form by others independently, but the combination here presented has worked so well and is so simple and cheap that it has seemed to merit the present brief notice.

Chicago, March 24th, 1877. NORMAN BRIDGE.

## Figures and Tables

**Figure f1:**